# Effects of Thermal Annealing on the Properties of Zirconium-Doped Mg_x_Zn_1−X_O Films Obtained through Radio-Frequency Magnetron Sputtering

**DOI:** 10.3390/membranes11050373

**Published:** 2021-05-20

**Authors:** Wen-Yen Lin, Feng-Tsun Chien, Hsien-Chin Chiu, Jinn-Kong Sheu, Kuang-Po Hsueh

**Affiliations:** 1 Department of Information Management, National Taichung University of Science and Technology, Taichung 40401, Taiwan; qqnice@nutc.edu.tw; 2 Institute of Electronics, National Yang Ming Chiao Tung University, Hsinchu 30010, Taiwan; ftchien@mail.nctu.edu.tw; 3 Department of Electronics Engineering, Chang Gung University, Tao-yuan 33302, Taiwan; hcchiu@mail.cgu.edu.tw; 4 Department of Photonics, National Cheng Kung University, Tainan 70101, Taiwan; jksheu@mail.ncku.edu.tw; 5 Department of Intelligent Production Engineering, National Taichung University of Science and Technology, Taichung 40401, Taiwan

**Keywords:** ZrO_2_, MgZnO, ZnO, MgO, thin film transistor, radio-frequency magnetron sputtering

## Abstract

Zirconium-doped Mg_x_Zn_1−x_O (Zr-doped MZO) mixed-oxide films were investigated, and the temperature sensitivity of their electric and optical properties was characterized. Zr-doped MZO films were deposited through radio-frequency magnetron sputtering using a 4-inch ZnO/MgO/ZrO_2_ (75/20/5 wt%) target. Hall measurement, X-ray diffraction (XRD), transmittance, and X-ray photoelectron spectroscopy (XPS) data were obtained. The lowest sheet resistance, highest mobility, and highest concentration were 1.30 × 10^3^ Ω/sq, 4.46 cm^2^/Vs, and 7.28 × 10^19^ cm^−3^, respectively. The XRD spectra of the as-grown and annealed Zr-doped MZO films contained Mg_x_Zn_1−x_O(002) and ZrO_2_(200) coupled with Mg(OH)_2_(101) at 34.49°, 34.88°, and 38.017°, respectively. The intensity of the XRD peak near 34.88° decreased with temperature because the films that segregated Zr^4+^ from ZrO_2_(200) increased. The absorption edges of the films were at approximately 348 nm under 80% transmittance because of the Mg content. XPS revealed that the amount of Zr^4+^ increased with the annealing temperature. Zr is a potentially promising double donor, providing up to two extra free electrons per ion when used in place of Zn^2+^.

## 1. Introduction

The use of zinc oxide (ZnO) as a semiconductor material in thin-film transistors (TFTs) has recently received attention because of the material’s excellent electrical characteristics, its usage of a low-temperature process, and its transparency performance in comparison with conventional silicon TFTs [1,2,3]. Therefore, ZnO TFTs are potential next-generation flat-panel display devices [4]. ZnO is a II–VI compound semiconductor that has several favorable characteristics, including wide energy bandgap (3.4 eV), large free exciton binding energy (60 mV), high carrier mobility, high transparency at room temperature, and excellent photoelectric, piezoelectric, and thermoelectric properties [3,4]. Additionally, the generation of hetero-structures is a basic requirement when developing structures for thin-film electronic and optoelectronic devices [3,4,5,6]. ZnO can be alloyed with MgO (bandgap = 7.8 eV) to form the ternary compound Mg_x_Zn_1−x_O (MZO), which has a larger energy band gap than ZnO and a detection spectrum that is thus located in a shorter wavelength region. Extending the usable wavelength range and improving the efficiency of quantum confinement structures are crucial tasks in band-gap engineering of MZO films [6,7,8,9].

MZO films have been grown homoepitaxially through pulsed-laser deposition, molecular beam epitaxy, and radio-frequency (RF) magnetron sputtering [9,10,11,12,13]. However, few studies have reported the thermodynamic stability of un-doped MZO films deposited through RF magnetron sputtering [12,13]. The fabrication of n- and p-type ZnO or MZO films through the element doping technique (doping with Fe, Ni, Ag, Mn, Cu, Al, Ga, or N) [14,15,16], which can reduce resistance and improve the electron mobility of MZO films, is critical for producing MZO-based optoelectronic and electronic devices. In this study, Zirconium (Zr) was selected as the dopant because it is readily available, has a comparable ionic size to Zn, and has the ability to act as a donor of an extra free electron per ion due to its four equatorial Zr (IV) metal ions [17,18]. We investigated the thermodynamic stability of Zr-doped MZO films through Hall measurements, X-ray diffraction (XRD), and X-ray photoelectron spectroscopy (XPS).

## 2. Experiment

Zr-doped MZO films were grown at room temperature on *c*-face sapphire substrates by using the RF sputtering method rather than through co-sputtering. The Zr-doped MZO films were formed using a 4-inch ZnO/MgO/ZrO_2_ (75/20/5 wt%) target made by GFE, Nürnberg, Germany). The materials in this target were mixed in powder and then were hot-pressed and sintered. The RF sputtering deposition conditions were identical to those in a previous report [9]. The RF power was 1 KW, the RF was conducted at 13.56 MHz, and an automatic impedance-matching network was employed; the total impedance of the circuit was regulated to 50 Ω, which is suitable for plasma ignition in typical sputtering environments. Before the deposition, the substrates were sequentially cleaned in an ultrasonic bath containing acetone, isopropyl alcohol, and deionized water for 5 min each. The distance between the target and the substrate was fixed at 7.2 cm, and the substrate was rotated at a constant speed of 20 rpm during sputtering. The chamber was purged with pure argon, and the target was pre-sputtered for 15 min before growth. Through the use of a mechanical pump and a cryopump, the chamber was initially evacuated to a base pressure of 2.0 × 10^−6^ Torr, and the pressure was sustained at 10 Torr during film deposition. The deposition conditions were as follows: argon flow rate of 9 sccm, working pressure of 1.2 × 10^−3^ Torr, and sputtering power of 150 W for the growth time of 45 min. The chamber was cooled using a water-cooled chiller system during the sputtering process. The thickness of the deposited Zr-doped MZO film was 600 nm, as measured using a Veeco/Dektak 6M profiler (Plainview, NY, USA). The Zr-doped MZO samples were then annealed at 700, 800, 900, and 1000 °C for 60 s in a nitrogen atmosphere in a rapid thermal annealing system (RTA, Premtek/ARTs 150, Hsinchu, Taiwan). This system employed tungsten halogen lamps (thermal increase rate ~80 °C/s) to increase the temperature, and the chamber was cooled using water and N_2_ gas (cooling rate ~1 °C/s). Additionally, a Hall effect measurement system (Quatek/HL550PC, Hsinchu, Taiwan) was used for determining various electrical parameters of the Zr-doped MZO films.

## 3. Results and Discussion

Table 1 presents the dependence of the carrier concentration, electrical sheet resistance, and Hall mobility of the Zr-doped MZO films on the annealing temperature. Hall measurement results for the as-deposited film are not shown because of the films’ high sheet resistance. For annealing temperatures >700 °C, the resistance decreased, whereas the concentration and mobility increased. The lowest resistance, highest mobility, and highest concentration were 1.30 × 10^3^ Ω/sq, 4.46 cm^2^/Vs, and 7.28 × 10^19^ cm^−3^, respectively, at an annealing temperature of up to 1000 °C. Table 1 shows that the carrier concentration and mobility increased with an increase in the annealing temperature. The experimental results revealed that thermal annealing considerably increased the number of oxygen vacancies and Zr^2+^ ions, which played the role of donor in the Zr-doped MZO films, resulting in an increase in conductivity [17,18]. Film quality and dopants can be improved through the out-diffusion of dopants or films re-composition induced by high thermal energy during annealing [19]. 

**Figure 1:**
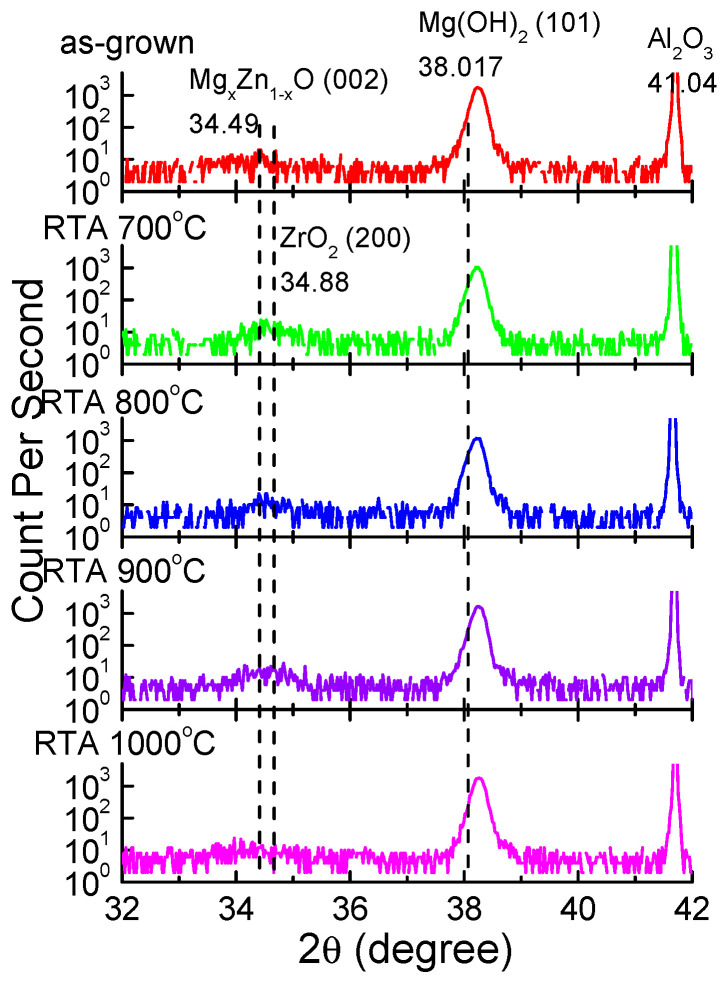
(Color Online) X-ray diffraction scan profiles of Zr-doped Mg_x_Zn_1−x_O (MZO) films annealed at various temperatures.

Figure 1 displays the XRD spectra (2θ-intensity scan) of the as-grown and annealed Zr-doped MZO samples. XRD was performed using a Siemens D5005-D XRD system and under Cu-K_α_ radiation (λ = 0.1542 nm). The XRD patterns of the Zr-doped MZO films were verified using the JCPDS Cards No. 36-1451, 44-1482, 45-0946, 27-0997, and 43-1484, and revealed Mg_x_Zn_1−x_O(002) and ZrO_2_(200) coupled with Mg(OH)_2_(101) at 34.49°, 34.88°, and 38.017°. The peaks of the Al_2_O_3_ sapphire substrate (JPCDS Card no. 27-0997) were observed at 41.04°. Similar XRD patterns were obtained at all annealing temperatures. The XRD curves in Figure 1 reveal the very weak diffraction peak of MgZnO(002) and ZrO_2_(200) and the strong diffraction peaks of Mg(OH)_2_(101) and Al_2_O_3_. The peak of Mg(OH)_2_ may be attributed to the reactions of MgO with H_2_O during the sputtering process. The intensity of the XRD peak near 34.88° decreased with temperature because the films that segregated Zr^4+^ from ZrO_2_(200) increased. Additionally, no large structural difference was discovered when the thermal treatments at 700, 800, 900, and 1000 °C were compared; however, differences in optical and electrical properties were observed.

**Figure 2:**
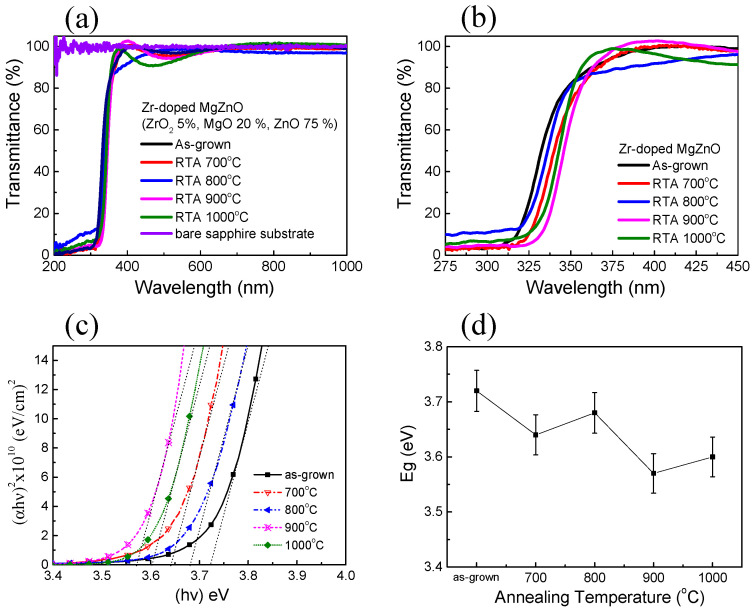
(Color Online) (**a**) Full transmission spectra and (**b**) transmission spectra in the 275–375 nm range of the as-grown and annealed Zr-doped MZO films deposited on sapphire substrates. (**c**) Relationship between (*αhν*)^2^ and photon energy *hν*. (**d**) Summary of the *E_g_* of the as-deposited and annealed Zr-doped MZO films.

Figure 2a shows the optical transmission of the Zr-doped MZO films, which was obtained by measuring the optical absorption edge shift by using a Hitachi U-4100 spectrophotometer. The transmittance spectra of Zr-doped MZO films were calibrated according to the transmittance spectra of a bare double-side polished sapphire substrate. The average transmission in the visible region (360–700 nm) was higher than 95%, and the spectrum had an absorption edge in the ultraviolet region (325–350 nm). Compared with related results for ZnO and Ga-doped ZnO films [19], the absorption edges of our Zr-doped MZO films were shifted toward the short wavelength at 348 nm under 80% transmittance because of their Mg content. Additionally, a promising approach is to combine MgO with ZnO by using a ZnO/MgO/ZrO_2_ (75/20/5 wt%) target to tailor the bandgap and crystal structure. Subsequently, the peak oscillation near the 400 nm wavelength in the curve was due to the irregular surface of the Zr-doped MZO films. Figure 2b shows the optical transmission of the Zr-doped MZO films in the 275–375 nm range, which enabled to determine detailed optical absorbance characteristics. The transmission of the films exhibited a relatively sharp absorption edge near 350 nm. The absorption edges of the annealed Zr-doped MZO films were observed at longer wavelengths at higher annealing temperature than those of the un-doped MZO films. However, in our earlier report, the absorption spectra of un-doped MZO films annealed at 700 and 800 °C contained two stages at wavelengths of 357 and 261 nm [9]. In the present study, the transmittance spectra revealed that the Zr-doped MZO films were more thermally stable than the un-doped MZO films when the thermal annealing temperature was high, which is consistent with the XRD results.

In a direct transition semiconductor, the absorption coefficient *α* and optical bandgap (*E_g_*) are related by the following equation: (*αhν*) = A(*hν −E_g_*)^1/2^, where A is a constant, *α* is the absorption coefficient, *hν* is the photon energy, and *E_g_* is the optical bandgap [20,21]. The *E_g_* value can be obtained by extrapolating the straight-line portion of the curve in Figure 2c to the photon energy axis. The energy bandgap of the as-grown Zr-doped MZO film was 3.72 eV. Figure 2d indicates that the energy bandgaps were 3.64, 3.68, 3.57, and 3.60 eV after annealing at 700, 800, 900, and 1000 °C, respectively. Such a slight decrease in optical bandgap energy generally occurs in annealed direct-transition-type semiconductor films [22]. Figure 2d shows that the *E_g_* curve was W-shaped and that *E_g_* generally decreased with an increase in annealing temperature. Because the XRD results indicated no considerable structural differences, we concluded that, first, the number of dopants, such as oxygen vacancies and Zr^2+^ ions, was slightly increased and, second, these dopants occupied the bandgap, causing a slight change in *E_g_*.

**Figure 3:**
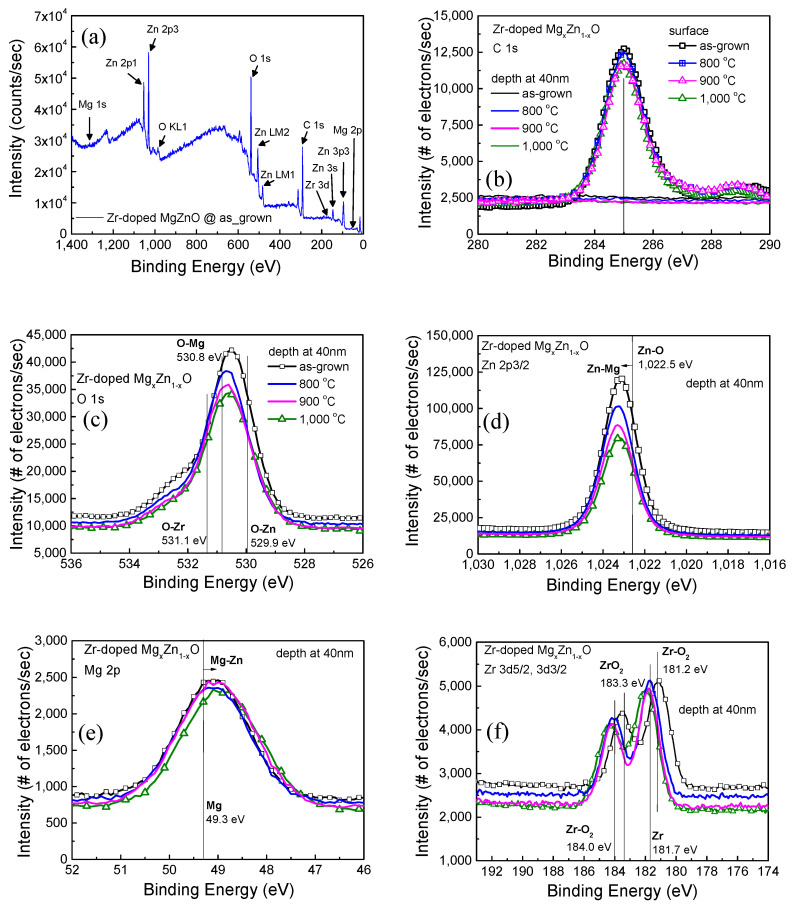
(Color Online) (**a**) X-ray photoelectron spectroscopy (XPS) spectrum of surface elements in the as-deposited Zr-doped MZO film. (**b**) C 1s, (**c**) O 1s, (**d**) Zn 2p3/2, (**e**) Mg 2p, and (**f**) Zr 3d XPS spectra of as-grown Zr-doped MZO films annealed at 800, 900, and 1000 °C, with spectra obtained at the surface and at a depth of 40 nm.

In this study, XPS was performed using a Thermo VG-Scientific/Sigma Probe instrument to investigate the composition of the as-grown and annealed Zr-doped MZO films. The carbon (C 1s) peak (at 285 eV) was employed as a reference in the alignment to binding energies [23]. The samples were etched to a 40 nm depth by an Ar ion beam for depth analysis in the same vacuum system after an XPS analysis of the surface. Figure 3 displays the C 1s, O 1s, Zn 2p3/2, Mg 2p, and Zr 3d spectra of the Zr-doped MZO films. The XPS spectrum of surface elements of the as-deposited Zr-doped MZO film is shown in Figure 3a. In the spectrum obtained after the film was etched to a depth of 40 nm, the C 1s spectrum peaks were not perceptible, as shown in Figure 3b, because the carbon contamination on the surface was removed. Figure 3c–f shows the O 1s intensity of the O–Zn/O–Mg/O–Zr binding energy, the Zn 2p3/2 intensity of the Zn–O binding energy, the Mg 2p intensity of the Mg–O binding energy, and the Zr 3d intensity of the Zr–O/Zr binding energy after the removal of carbon surface contamination. Compared with those in the spectra of the as-grown film and the film annealed at 1000 °C, the O 1s, Zn 2p, and Mg 2p intensities are lower (Figure 3c,d and Figure 3e, respectively). The O 1s spectra revealed that the O–Zr, O–Mg, and O–Zn binding energies were slightly lower after thermal annealing at 1000 °C (Figure 3c,d). The Mg 2p spectrum at 49.3 eV for the composition of Mg only had a slightly lower intensity and a shift to the composition Mg–Zn. These results indicated that O–Zn and O–Mg were released and combined to form Mg–Zn, resulting in more oxygen vacancies.

The Zr 3d spectrum presented in Figure 3f indicates that the Zr–O_2_ binding energy (181.2 eV) was shifted to the Zr^2+^ ion binding energy (181.7 eV) after thermal annealing at 1000 °C. The oxygen number of vacancies and the degree of Zr segregation were greater at higher annealing temperatures, which (1) promoted the formation of neutral, (2) increased concentration and mobility, and (3) decreased resistance. Stephania et al. selected zirconium as a dopant in ZnO and MgZnO because of its abundance, its comparable ionic size to Zn, and its role as a double donor, providing up to two extra free electrons per ion when used in place of Zn^2+^ [17,24]. The close ionic sizes of Zr^4+^ and Zn^2+^ [25] (i.e., 0.745 Å for Zr and 0.740 Å for Zn) are expected to help minimize lattice distortion [17,18]. Thus, the number of Zr^2+^ ions and oxygen vacancies can be inferred to be higher after thermal annealing at 1000 °C; this results in higher carrier concentration and, in turn, lower resistance.

## 4. Conclusions

This study reports the thermal and optical properties of Zr-doped MZO films that were deposited through RF magnetron sputtering. The intensity of the XRD peak near 34.88° decreased with temperature because the films that segregated Zr^4+^ from ZrO_2_(200) increased. The Zr-doped MZO films were experimentally verified to achieve high transmittance (95% in the visible region). The absorption edges of the Zr-doped MZO films were shifted toward the short wavelength of 348 nm under 80% transmittance because of their Mg content. The energy bandgaps were 3.64, 3.68, 3.57, and 3.60 eV after annealing at 700, 800, 900, and 1000 °C, respectively. Additionally, XPS analysis of the as-grown and annealed Zr-doped MZO films indicated that Zr^4+^ is suitable as the dopant in ZnO and MgZnO because it can act as a double donor, providing up to two extra free electrons per ion when used in place of Zn^2+^. The XPS results are consistent with the Hall results.

## Figures and Tables

**Table 1:** Hall measurement results for the as-deposited and annealed Zr-doped Mg_x_Zn_1−x_O films.

Temperature	Sheet Resistance Ohmic/sq	Mobility cm^2^/Vs	Carrier Concentration/ cm^3^
As-deposited	N.A.	N.A.	N.A.
700 °C/N_2_/60 s (RTA700°C)	1.59 × 10^5^	0.881	−3.04 × 10^18^
800 °C/N_2_/60 s (RTA800°C)	5.38 × 10^4^	1.61	−4.90 × 10^18^
900 °C/N_2_/60 s (RTA900°C)	2.45 × 10^3^	3.70	−4.67 × 10^19^
1000 °C/N_2_/60 s (RTA1000°C)	1.30 × 10^3^	4.46	−7.28 × 10^19^

## Data Availability

Not applicable.
